# Challenges in cataract surgery after penetrating keratoplasty managed using femtosecond laser: A series of 3 case reports

**DOI:** 10.1097/MD.0000000000038614

**Published:** 2024-06-21

**Authors:** Qiaomei Tang, Ke Yao

**Affiliations:** a Eye Center, The Second Affiliated Hospital, Zhejiang University School of Medicine, Hangzhou, China; b Zhejiang Provincial Key Lab of Ophthalmology, Hangzhou, China.

**Keywords:** case report, cataract, femtosecond laser, penetrating keratoplasty

## Abstract

**Introduction:**

Cataract surgery in patients after penetrating keratoplasty (PKP) is often challenging because of changes in corneal structure induced by PKP and primary corneal disease. Femtosecond laser-assisted cataract surgery offers several advantages over conventional phacoemulsification, and has been widely used in complicated cataract surgery.

**Case report:**

We report the use of femtosecond laser-assisted cataract surgery in 3 challenging cases after penetrating keratoplasty. Case 1 involved a patient with hard nuclear grade IV° cataract. After surgery, his corrected distance visual acuity (CDVA) improved from 20/400 to 20/25, and the endothelial cell loss (ECL) % was 12.05 % at 3 months postoperatively. The rotation of the toric IOL in Case 1 was 2°. Case 2 involved a patient with severe nuclear cataract and an endothelial cell density of 837 cells/mm^2^. After surgery, the CDVA improved from 20/100 to 20/40. The ECL% was 4.06% at 1 week postoperatively. Case 3 was a 91-year-old woman with a short axis length of 21.35 mm and an endothelial cell density number of 1238 cells/mm^2^. After surgery, the CDVA improved from light perception to 20/133, and the ECL% was 26.09% at 1 week postoperatively; ECL% was 2.67% at 1 month post-operation. The corneal grafts were transparent.

**Conclusion:**

Femtosecond laser-assisted cataract surgery seems to be an effective, predictable, and safe approach for challenging patients after PKP, and improves visual recovery and optimal refractive outcomes.

## 1. Introduction

Previous studies have reported that 24% to 60% of the patients after a single penetrating keratoplasty (PKP) developed cataract formation.^[1]^ Cataract surgery after PKP may cause corneal graft rejection and decompensation, and the rate of corneal endothelial loss caused by phacoemulsification after PKP is much higher than that in non-keratoplasty eyes.^[2]^ Previous studies reported a mean endothelial cell loss ranging from 4.2% to 7.8% per year in patients after PKP.^[2]^ Complex corneal diseases and postoperative steroid drugs often lead to hard cataracts. Scarring around the edge of the corneal graft may obscure subsequent cataract surgery. Preoperative primary disease and postoperative intraocular inflammation of the PKP often cause posterior pupil adhesion or anterior iris adhesion.^[3]^ Therefore, cataract surgery after PKP is often challenging because of changes in corneal structure induced by PKP and primary corneal disease.

Femtosecond lasers represent a promising new technology for cataract surgery. Femtosecond laser-assisted cataract surgery (FLACS) offers several advantages over the conventional phacoemulsification. FLACS has 4 main functions: creation of a consistently sized round capsulotomy, treatment of keratometric astigmatism with arcuate incisions, construction of clear corneal incisions, and fragmentation of the lens.^[4]^ It has been reported that FLACS can reduce the amount of phacoemulsification energy delivered by 33% to 70% by softening the lens. Recent studies have shown that FLACS significantly reduces phacoemulsification energy, which may result in less endothelial damage and reduce the risk of postoperative corneal decompensation and bullous keratopathy, particularly in patients after PKP.^[5]^ Here, we applied FLACS to 3 challenging patients with cataract formation after PKP.

## 2. Patient information

### 2.1. Case 1 – a patient with a hard nuclear, grade IV° cataract

**Figure 1.**
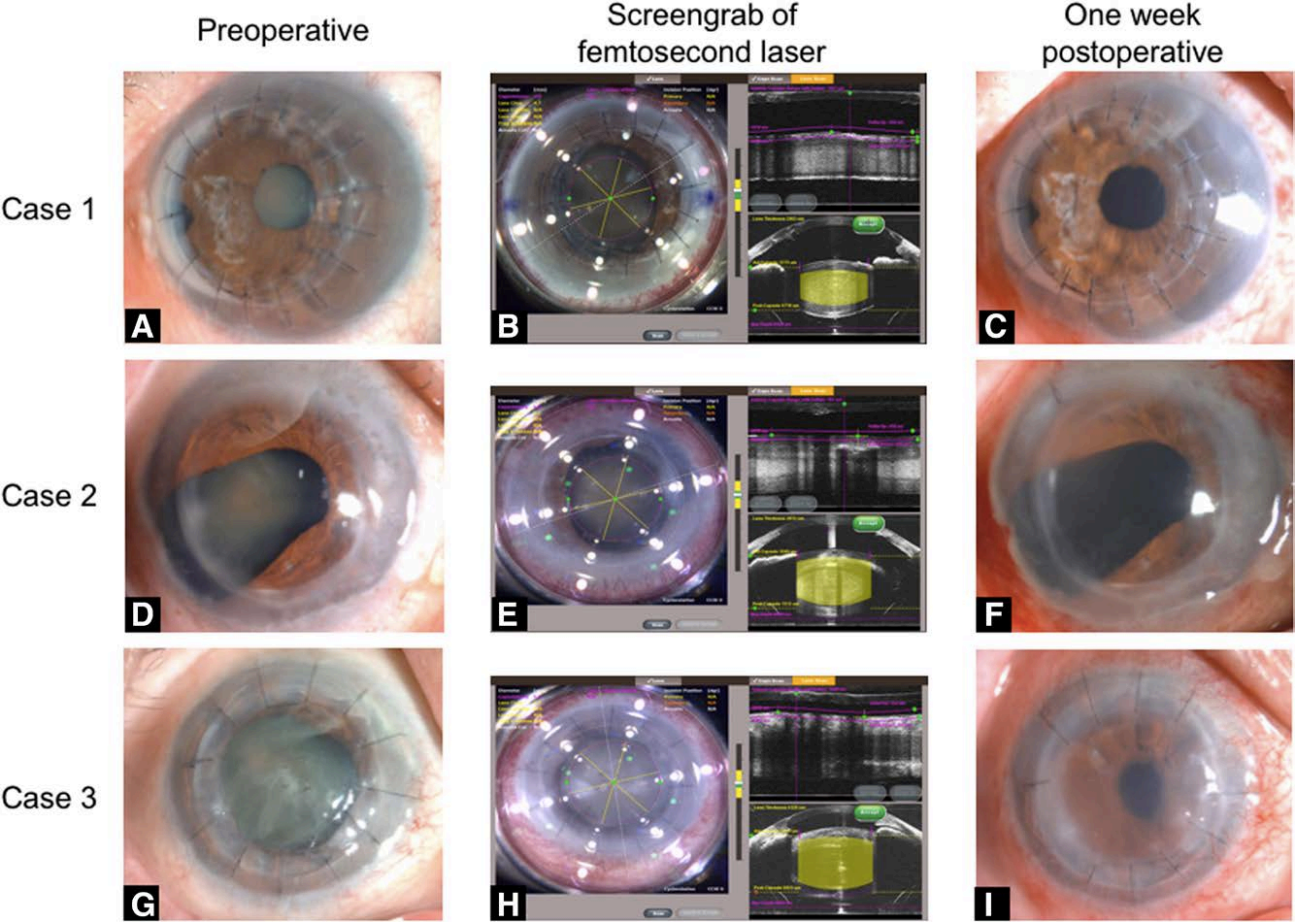
Anterior segment photography showing preoperative images of case 1 (A), case 2 (D), and case 3 (G); capsulorhexis performed with the femtosecond laser and a sextant chop pattern used for lens fragmentation of case 1 (B), case 2 (E), and case 3 (H); 1 wk postoperative images of case 1 (C), case 2 (F), and case 3 (I).

The patient was a 71-year-old man with severe cortical and hard nuclear opacities of the lens, C_4_N_3_P_2_ (Lens Opacities Classification System II, LOCS II) IV° hard nucleus (Emery-Little classification) (Fig. 1A). He complained of a progressive decrease in vision in his left eye, which had penetrating keratoplasty 2 years ago because of viral keratitis with perforation. The patient subsequently developed a hard cataract with high post-keratoplasty astigmatism. Corrected distance visual acuity (CDVA) was 20/400. Corneal topography using Scheimpflug imaging (Pentacam; Oculus Optikgeräte GmbH, Wetzlar, Germany) showed high astigmatism with an asymmetrical bowtie pattern and a central corneal thickness (CCT) of 527 µm. Keratometric values (K1/K2) were 43.1/47.5 D @ 77.3. Biometric data (axial length, anterior chamber depth, and lens thickness) were measured using an optical biometer (IOL Master 700; Carl Zeiss Meditec, Jena, Germany), and ultrasonography pachymetry and biometry (Cinescan; Quantel Medical, Cournon d’Auvergne, France). The IOL spherical and cylinder powers and the intended axis were calculated using the IOL manufacturer’s online calculator (www.TecnisToricCalc.com). The surgically induced astigmatism was assumed as 0.3 D at the incision axis. The patient was diagnosed with complicated cataract (hard core) in the left eye and left eye after penetrating keratoplasty.

#### 2.1.1. Therapeutic intervention

A femtosecond laser platform (Alcon-LenSx, Aliso Viejo, CA, USA) was used to perform this surgery.^[6]^ The same experienced surgeon (KY) performed all surgical procedures. The patient was seated and staring at a distant target in both eyes. The limbus was marked at 3 and 9 o’clock positions using a slit lamp before entering the operating room. The corneal scar was peripheral to the site of the planned capsulotomy. A 4.8-mm capsulorhexis was performed using a femtosecond laser and a sextant chop pattern was used for lens fragmentation (Fig. 1B). After femtosecond laser pretreatment, the surgery was completed using a standard phacoemulsification procedure (Stellaris system; Bausch & Lomb, New York, USA). A 0.8-mm side-port incision and a 2.2 mm corneal incision were made manually using a keratome according to the preset position. The effective phacoemulsification time (EPT) was 9 seconds and the total phacoemulsification time was 1 minute 4 seconds. A customized toric IOL (TECNIS^®^ TORIC, ZCT400, +20.0 D) was implanted into the capsular bag. The IOL alignment was checked and ensured. The corneal wound was hydrated and the final IOL axial orientation was recorded using a digital video clip. All patients were prescribed topical levofloxacin 0.5% 4 times for 1 day before surgery. Pupillary dilation was achieved with the instillation of 1 drop of tropicamide every 15 minutes 3 times. After surgery, topical dexamethasone tobramycin 4 times a day for 2 weeks and pranoprofen 4 times a day for 1 month were administered to all patients.

#### 2.1.2. Follow-up and outcomes

On postoperative day 1, the CDVA was 20/33 with almost no corneal edema, which improved to 20/25 over the next 3 months. Subjective refraction was stable in the 1.00 D sphere and 3.00 D cylinder at the 3-month follow-up. Endothelial cell loss (ECL) was calculated as the percentage of the preoperative endothelial cell density (ECD). The preoperative ECD numbers were 2963 cells/mm^2^ and 2629 cells/mm^2^ at 1 week and 2606 cells/mm^2^ at 3 months after surgery, respectively. The ECL had decreased by 12.05% 3 months postoperative. Anterior segment photography showed a transparent cornea at 1 week post-operation (Fig. 1C). Postoperative toric IOL rotation was also assessed. The toric IOL was centered in the bag and oriented at 76°; the postoperative rotation was 2° at 3 months postoperatively. The corneal graft was transparent 1 year after femtosecond laser-assisted cataract surgery.

### 2.2. *Case 2 – a patient with a severe nuclear cataract and a ECD number of 837 cells/mm^2^*

The patient was a 76-year-old man with cataracts, C_3_N_3_P_2_ (LOCS II classification), and III° nuclear hardness (Emery-Little classification) (Fig. 1D). The patient had a lower ECD count 837 cells/mm^2^. He complained of blurred vision in his right eye, which had undergone penetrating keratoplasty 29 years previously because of a corneal ulcer. He subsequently developed a cataract 3 years ago. The CDVA was 20/100. Slit-lamp examination revealed a clear corneal graft and a severe nuclear opacity of the lens. The corneal topography showed high astigmatism with an asymmetrical bowtie pattern and a CCT of 570 μm. The keratometric values (K1/K2) were 44.9/54.1 D @ 46.5. The ECD count was 837 cells/mm^2^. Biometric data were measured using optical biometer, ultrasonography pachymetry, and biometry. Fundus examination results were normal. The patient was diagnosed with complicated cataract in the right eye and right eye after penetrating keratoplasty.

#### 2.2.1. Therapeutic intervention

The same experienced surgeon (KY) performed all surgical procedures. A 5.0-mm capsulorrhexis was performed using a femtosecond laser and a sextant chop pattern was used for lens fragmentation (Fig. 1E). The EPT was 8 seconds and the total phacoemulsification time was 1 minute 5 seconds. An IOL (CT ASPHINA 509M, +16.0 D) was implanted in the capsular bag.

#### 2.2.2. Follow-up and outcomes

On postoperative 1 week, the CDVA was 20/40 with slight corneal edema (Fig. 1F). The postoperative ECD number was 803 cells/mm^2^ 1 week after surgery. The ECL% was 4.06%. The corrected distance visual acuity was 20/33 3 months after surgery.

### 2.3. *Case 3 – a 91-year-old woman with a short axis length of her right eye and an ECD number of 1238 cells/mm^2^*

A 91-year-old woman complained of blurred vision in her right eye, which had undergone penetrating keratoplasty 2 years previously because of corneal dystrophy. She later developed a cataract, C_4_N_2_P_3_ (LOCS II classification) II° nuclear hardness (Emery-Little classification) (Fig. 1G). The CDVA was light perception. The left eye had been blind due to glaucoma for ten years. Corneal topography showed astigmatism with an asymmetrical bowtie pattern and CCT of 533 μm. The keratometric values (K1/K2) were 41.0/43.5 D @ 144.8. The ECD numbers were 1238 cells/mm^2^. The axis length of the patient’s eye was very short at only 21.35 mm. The patient was diagnosed with complicated cataract in the right eye and right eye after penetrating keratoplasty.

#### 2.3.1. Therapeutic intervention

The same experienced surgeon (KY) performed all surgical procedures. A 5.0-mm capsulorhexis was performed using a femtosecond laser, and a sextant chop pattern was used for lens fragmentation (Fig. 1H). The EPT was 10 seconds and the total phacoemulsification time was 1 minute and 16 seconds, respectively. An IOL (TECNIS ZCB00, +30.0 D) was implanted in the capsular bag.

#### 2.3.2. Follow-up and outcomes

On postoperative 1 week, the CDVA was 20/133 with slight corneal edema (Fig. 1I). postoperative ECD number was 905 cells/mm^2^ at 1 week after surgery, and the ECL% was 26.90%. The ECD number was 1205 at 1 month post-surgery, and the ECL% was 2.67%.

## 3. Discussion

FLACS has provided a technique for reproducible precision in cataract surgery, especially for optimizing premium intraocular lens performance and meeting the high expectations of patients in the era of refractive cataract surgery. It is also a useful tool to decrease the risk of intraoperative and postoperative complications in potentially complex cases. Here, we successfully performed femtosecond laser-assisted cataract surgery in 3 challenging cases after PKP, with no postoperative complications. Case 1 involved a patient with hard nuclear grade IV° cataract. Case 2 was a patient with a severe nuclear cataract and an ECD number of 837 cells/mm^2^. Case 3 was a 91-year-old woman with a short axis length of 21.35 mm and an ECD number of 1238 cells/mm^2^. All 3 patients showed improved visual acuity after surgery and were highly satisfied. The detailed intraoperative parameters of the 3 cases was listed in Table 1.

**Table 1:** Intraoperative parameters of the 3 cases.

Parameters	US^a^ power, %	EPT^b^, s	ECD^c^ % (1 wk postoperative)
Case 1	14%	9′	11.27%
Case 2	12.8%	8′	4.06%
Case 3	12.5%	10′	26.90%

a US power = ultrasound power.

b EPT = effective phacoemulsification time.

c ECD = endothelial cell loss.

The 2 critical steps for successful cataract surgery in our cases were obtaining a central continuous capsular capsulotomy and minimizing the ultrasound energy. Some studies have demonstrated less IOL decentration after FLACS than after manual CCC.^[7]^ This becomes extremely important when implanting premium intraocular lenses, as the lens position is of increased importance to the lens’s success. The most important parameters of toric IOLs for accurate alignment and rotational stability are the size and concentration of the anterior capsulotomy site. The precise intraoperative alignment and rotational stability of the toric IOL provide excellent correction of astigmatism.^[8]^ Every degree of off-axis rotation of toric IOLs leads to a reduction of approximately 3.3% in IOL cylinder power. Its optimal size, achieved by femtosecond laser-assisted capsulotomy, ensures a 360° overlap of the anterior remaining capsule with the IOL optic, improving rotational stability and minimizing IOL tilt.^[7]^ In case 1, a toric IOL (ZCT400, +20.0 D) was implanted into the patient to correct for high astigmatism after PKP. The toric IOL was centered in the bag and oriented at 76 degrees at 3 months post-operation, and the postoperative rotation was 2°. Femtosecond laser-assisted capsulotomy ensures precise intraoperative and postoperative alignment and rotational stability of the toric IOL in eyes after PKP. In patients undergoing cataract surgery after corneal transplantation, toric IOL implantation should be considered if the conditions are appropriate. In eyes with shallow ACD, FLACS is a safe and effective technique that significantly reduces postoperative ECL and corneal inflammation compared with conventional phacoemulsification.^[9]^

It is important to minimize damage to endothelial cells in patients after PKP during cataract surgery. FLACS has been designed to prefragment the lens using different patterns to segment the nucleus or soften harder cataracts with the intention of reducing the thermal or ultrasound energy delivered to the corneal endothelium.^[4]^ In our previous study, the ECL was <31% in the conventional phacoemulsification cataract surgery on the first day after surgery and 19.96% at 3 months postoperatively, and in the FLACS was only 7.85% at the last examination. In our 3 cases after PKP, the ECL% in case 1 with a hard nuclear cataract, graded IV° was 12.5% at 3 months postoperatively. The ECL% in case 3 with a short axis length and a lower ECD number was 26.9% at 1 week post-operation and 2.67% at month post-operation. The ECL% in Case 2 was 4.06% at 1 week postoperatively. Owing to the use of a femtosecond laser to liquefy the nucleus, a very small ultrasound is required to remove the lens and minimize endothelial cell damage in patients after PKP. However, this issue should be considered over a long follow-up period.

In addition, scarring around the edge of the corneal graft may obscure subsequent cataract operations, leading to intraoperative complications such as incomplete femtosecond laser capsulorhexis. It can be treated with anterior staining and manual capsulorhexis. Another limitation is that femtosecond laser-assisted capsulotomy is not suitable for patients with pupillary synechiae and incomplete pupil dilation during preoperative mydriasis.

In conclusion, the use of a femtosecond laser allowed controlled capsulorhexis and minimized the ultrasound energy required to emulsify the nucleus, thereby protecting endothelial cells in postoperative transplant corneas. Femtosecond laser-assisted cataract surgery seems to be an effective, predictable, and safe approach for challenging patients after PKP, and improves visual recovery and optimal refractive outcomes.

## Author contributions

**Data curation:** Qiaomei Tang.

**Formal analysis:** Qiaomei Tang.

**Investigation:** Qiaomei Tang.

**Methodology:** Qiaomei Tang.

**Project administration:** Qiaomei Tang, Ke Yao.

**Writing – original draft:** Qiaomei Tang.

**Conceptualization:** Ke Yao.

**Supervision:** Ke Yao.

**Validation:** Ke Yao.

**Writing – review & editing:** Ke Yao.
